# Teaching, assessment and best practice in undergraduate psychiatry education in the UK: cross-sectional survey

**DOI:** 10.1192/bjb.2024.2

**Published:** 2024-12

**Authors:** Deepika Sharma, Thomas Hewson, Sridevi Sira Mahalingappa, James Fallon, Declan Hyland, Seri Abraham, Alexa Sidwell, Subodh Dave

**Affiliations:** 1 Princess Alexandra Hospital NHS Trust, Harlow, UK; 2 University of Manchester, Manchester, UK; 3 Pennine Care NHS Foundation Trust, Ashton-under-Lyne, UK; 4 South London and Maudsley NHS Foundation Trust, London, UK; 5 University of Nottingham, Nottingham, UK; 6 Brighton and Sussex Medical School, Brighton, UK; 7 Liverpool Medical School, Liverpool, UK; 8 Derbyshire Healthcare Foundation Trust, Derby, UK; 9 University of Bolton, Bolton, UK; 10 Royal College of Psychiatrists, London, UK

**Keywords:** Education and training, undergraduate education, psychiatry curriculum, medical school, medical undergraduate

## Abstract

**Aims and method:**

We conducted a cross-sectional survey to examine how undergraduate psychiatry is taught and assessed across medical schools in the UK that have at least one cohort of graduated students.

**Results:**

In total, 27 medical schools completed the survey. Curriculum coverage of common mental disorders, assessment skills and mental health law was broadly consistent, although exposure to psychiatric subspecialties varied. Significant variation existed regarding the duration of psychiatry placements and availability of enrichment activities. Small-group teaching, lectures and e-learning were the most frequent teaching modalities and various professionals and lived experience educators (patient and/or carers) contributed to teaching. Objective structured clinical examinations and multiple-choice questions dominated assessments.

**Clinical implications:**

Medical schools should consider increasing students’ exposure to different psychiatric subspecialties and integrating physical and mental health training to address comorbidity and promote holistic care. Future research should explore whether specific undergraduate experiences promote greater career interest and skills in psychiatry.

The Global Burden of Disease Study in 2019 showed that mental disorders are among the top ten leading causes of disease burden worldwide.^1^ Furthermore, in England, the latest UK Adult Psychiatric Morbidity Survey (in 2014) found that 1 in 6 adults met criteria for a common mental disorder. This figure is higher in general hospital in-patient settings, where increased rates of depression, anxiety, delirium and dementia have all been demonstrated.^2^ It is therefore important that all doctors receive adequate training in psychiatry, since all doctors are likely to encounter patients needing mental health support. For many doctors, psychiatry teaching mostly takes place at undergraduate level. In the UK, fewer than half of newly qualified doctors undertake psychiatry placements as part of the UK Foundation Programme.^3,4^

As of 2021, 45 universities in the UK offered undergraduate medical degree programmes and 9 were being reviewed to award medical degrees.^5^ UK medical schools must meet uniform standards set by the General Medical Council (GMC), although individual institutions determine their own course structure, clinical placements, and styles of teaching and assessment.^6^ Karim et al previously mapped the delivery of the undergraduate psychiatry curriculum across 27 UK medical schools in 2009.^7^ They found significant variability and recommended more work to reach consensus on what constitutes the ‘ideal curriculum’.^7^ Since then, comprehensive guidance has been provided by the Royal College of Psychiatrists (RCPsych), GMC and World Federation for Medical Education regarding best practice in undergraduate medical and psychiatric education.^6,8–10^ The introduction of a Medical Licensing Assessment (MLA) by the GMC in 2024 will likely promote a more standardised curriculum across medical schools.^11^

Considering these changes to the medical school landscape, we aimed to explore how undergraduate psychiatry training is currently being delivered across the UK.

## Method

Ethical approval for this research was granted by the Faculty of Medicine & Health Sciences Research Ethics Committee at the University of Nottingham on 7 May 2021 (approval number FMHS 245-0421). All participants gave written consent for study involvement and publication of their anonymised data.

The project team designed a structured survey exploring the content and delivery of undergraduate psychiatry teaching and assessment. The survey was based on a previous survey by Karim et al^7^ and encapsulated four key domains highlighted in the RCPsych's *Choose Psychiatry: Guidance for Medical Schools* report: excellence in teaching, quality placements, leadership and enrichment activities.^8^

The survey was hosted on Qualtrics XM and split into five key sections:^12^

- details of each university's medical course
- features of psychiatry placements, including their duration, content and teaching methods used
- availability of psychiatry teaching throughout the curriculum
- assessments in psychiatry
- curriculum changes due to COVID-19 and/or the upcoming MLA.

A mixture of closed and free-text questions were used to provide quantifiable measures and exploration of differences between medical schools. The survey questions are shown in Supplementary Appendix 1, available at https://dx.doi.org/10.1192/bjb.2024.2.

Pilot testing across three UK medical schools ensured content and face validity of the survey. The final version was electronically distributed via email to undergraduate psychiatry leads identified through the RCPsych's undergraduate forum during August 2021. Undergraduate leads were given 8 weeks to respond. Two additional emails were sent to non-responders 6 and 8 weeks after the initial email, to increase participation rates.

Ten medical schools had been in existence for less than 5 years and these were excluded from the sample on the grounds that these courses had not had students progress to graduation. Furthermore, it was felt that newer medical schools might have not fully scoped out their curricula, operationalised their teaching and assessments or collaborated with students to establish enrichment psychiatry activities at the time of the survey.

Quantitative data were analysed, such as the proportion of medical schools using specific teaching or assessment methods. All proportions and percentages were calculated based on the total number of medical schools responding to each individual question.

Qualitative data were exported into NViVo 12 for Mac for inductive thematic analysis.^13^ Each participant's responses were coded according to their content and meaning, and codes were grouped together to create themes. The analysis was independently completed by two authors (D.S. and T.H.), who compared their results for similarities and differences. The final themes were agreed by both authors and interrogated by a third author (S.S.M.).

## Results

### Respondents

Of the 35 eligible UK medical schools, 27 (77%) responded to the survey: 21 from England, 4 from Scotland and 2 from Wales.

### Clinical placements

**Fig. 1:**
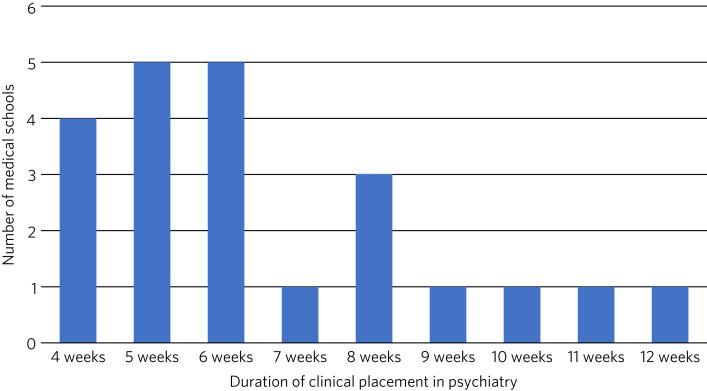
Duration of clinical placements in psychiatry offered across 27 UK medical schools in 2021.

The length of clinical placements during the psychiatry module varied from 4 to 12 weeks, with 5 or six 6 weeks being most common (*n* = 10) (Fig. 1).

### Teaching methods and roles

All the medical schools utilised small-group teaching. Beyond this, the most used modalities to deliver psychiatry curricula were: lectures (89%), e-learning (85%), case-based learning (85%), simulation (77%) and expert-patient teaching sessions (69%). Case-based learning involves using authentic clinical cases to teach students how to apply theoretical knowledge to clinical practice.^14^ Some medical schools conducted student-led teaching initiatives (42%), journal clubs (32%) and film clubs (24%). Very few institutions utilised webinars prior to the COVID-19 pandemic (13%), and few used virtual reality (12.5%), book clubs (12%) or gaming (5%) to teach psychiatry. There was considerable variation in the time allocated to these activities (Supplementary Table 2, Appendix 2). The median amount of time spent on small-group psychiatry teaching was 11–20 h during mandatory psychiatry modules, compared with 6–10 h for lectures and e-learning and 3–5 h for case-based learning, simulation and workshops. The median amount of time spent on expert-patient educator sessions was 1–2 h.

All universities had psychiatrists in appointed teaching roles to deliver psychiatry teaching (*n* = 27) and clinical academics were appointed in most schools (*n* = 19). Other appointed roles to teach psychiatry included expert-patient educators (*n* = 10), nurses (*n* = 9), pharmacists (*n* = 9), psychologists (*n* = 8), general practitioners (*n* = 7), non-clinical staff (*n* = 6), advanced clinical practitioners (*n* = 3) and occupational therapists (*n* = 3). One medical school reported employing physician associates (*n* = 1) and another reported involving carers in delivering psychiatry teaching (*n* = 1).

### Undergraduate psychiatry curricula

Large variation existed regarding the coverage of psychiatric subspecialties, but all medical schools taught generic skills in psychiatry, such as history taking and mental state examination. Coverage of common mental disorders (e.g. psychotic, affective and personality disorders) was largely consistent across medical schools (Table 1).

**Table 1:** The proportion of UK medical schools (*n* = 27) covering specific curricula content during mandatory psychiatry placements in 2021

Proportion of medical schools	Curriculum topics covered during mandatory psychiatry placements^a^
100%	Depression (*n* = 27) History taking (*n* = 27) Mental state exam (*n* = 27) Personality disorders (*n* = 27) Psychopharmacology (*n* = 27) Psychotic disorders (*n* = 27) Self-harm (*n* = 27)
75–99%	Addictions (*n* = 24) Anxiety disorders (*n* = 26) Child and adolescent psychiatry (*n* = 22) Dementia (*n* = 25) Eating disorders (*n* = 21) Intellectual disabilities (*n* = 23) Mental health legislation (*n* = 26) Mood disorders (*n* = 26) Older adult (*n* = 24) Post-traumatic stress disorder (*n* = 22) Risk assessment (*n* = 25)
50–74%	Careers in psychiatry (*n* = 15) Communicating with acutely distressed patients (*n* = 16) Confidentiality (*n* = 16) Dual diagnosis (*n* = 19) Forensic psychiatry (*n* = 14) Liaison psychiatry (*n* = 10) Perinatal psychiatry (*n* = 19) Physical health emergencies in psychiatry (*n* = 18) Professionalism (*n* = 15) Stigma in psychiatry (*n* = 18)
25–49%	De-escalation skills (*n* = 13) Dissociative disorders (*n* = 9) Neuropsychiatry (*n* = 11) Social justice/deprivation (*n* = 9)
0–24%	Ethnicity and mental health (*n* = 6) Public mental health (*n* = 5) Sleep disorders (*n* = 6) Spirituality in psychiatry (*n* = 5) Technological advances in psychiatry (*n* = 4)

a. Mandatory psychiatry placement refers to the main psychiatry placement that all students must undertake as part of the undergraduate medical degree programme.

All the medical schools provided undergraduates with placements on in-patient psychiatric wards and most described providing exposure to community psychiatry (*n* = 21). Most (22/27) medical schools reported offering students dedicated space for reflection on clinical encounters, most commonly through Balint groups (*n* = 11), reflective practice sessions (*n* = 6) or other non-specified means (*n* = 5).

Most medical schools integrated psychiatry teaching into that of other medical specialties, for example delivering child and adolescent mental health teaching during the paediatric module/placement or covering postnatal depression during the women's health module. Psychiatry was most commonly taught alongside general practice and paediatrics (*n* = 19). Psychiatry teaching was also incorporated into modules on geriatric medicine (*n* = 15), women's health (*n* = 13), neurology (*n* = 10), emergency medicine (*n* = 8), gastroenterology (*n* = 7), cardiology (*n* = 4), genitourinary medicine (*n* = 2), respiratory medicine (*n* = 2) and surgery and anaesthetics (*n* = 2).

### Assessments

**Fig. 2:**
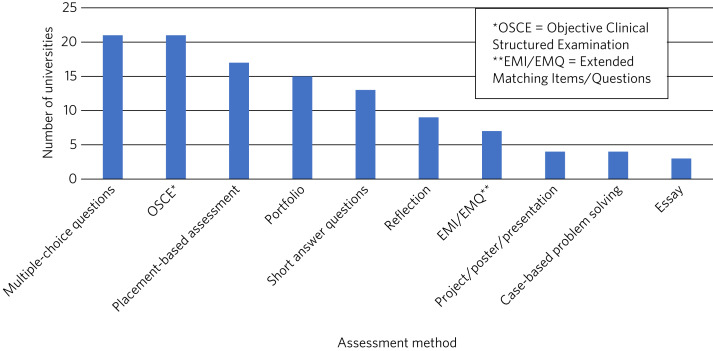
Number of UK medical schools (*n* = 27) utilising various assessment methods in psychiatry in 2021.

Numerous assessment methods were used to ascertain competence in psychiatry, with the most popular being multiple-choice questions and objective structured clinical examinations (OSCEs) (Fig. 2).

### Quality assurance and intra-university variation

Ten medical schools highlighted that individual students’ exposure to psychiatry varied. This was often attributed to geographical differences in the availability of psychiatric subspecialties across different regions where students undertake their placements. Two medical schools described implementing quality assurance systems to ensure equivalent learning experiences for all undergraduates; these involved convening regular meetings with all psychiatry placement leads at different sites and visiting students on placements weekly:

‘We aim for some exposure to old age, child, outpatient, addiction, but not all students get that ideal’ (Respondent 14).

### Extra-curricular opportunities

In total, 23 (92%) medical schools reported offering optional psychiatry-related modules; 2 (8%) confirmed that such opportunities were not available at their institution. These optional modules spanned various topics, including specific mental disorders (e.g. psychosis), prescribing (e.g. attitudes towards benzodiazepines), psychoactive substances (cannabis), reporting (media and mental health) and arts-based topics (creativity and mental illness). Some medical schools reported offering ‘one’ or only a few of these optional psychiatry modules, whereas others described providing ‘a large range’:

‘We offer student-selected components in psychiatry and try to facilitate any topics that the students are interested in’ (Respondent 2).

In free-text responses, seven medical schools described providing additional clinical exposure in psychiatry for interested undergraduates as part of ‘shadowing placements’, ‘experience weeks’ or ‘extended clinical placements’. Other extra-curricular opportunities reported included attending grand rounds and Schwartz rounds in psychiatry; film or journal clubs; alcoholics anonymous (AA) meetings; and observing psychiatry on-call shifts. Some universities (*n* = 5) described offering audit, quality improvement or research projects in psychiatry for interested students. Some medical schools referenced the Psychiatry Early Experience Programme (PEEP), where first- and second-year undergraduates shadow core trainees in psychiatry.^15^

### Educational impacts of COVID-19

Twenty-five medical schools described implementing changes to their curricula during the COVID-19 pandemic. Ten 10 respondents referred to clinical psychiatry rotations being suspended, shortened or ‘simplified’ during the pandemic; conversely, four respondents commented that the quantity and/or content of psychiatry teaching did not significantly change at their institutions.

Twenty-five medical schools described transitioning to online methods and reported using Zoom, Blackboard Collaborate or MS Teams to deliver teaching and assessment online. Examples of online teaching methods included virtual psychiatry clinics, remote multidisciplinary team meetings, clinical skills videos, online lectures and virtual simulation training with actors:

‘At the start of the pandemic we put in a lot of work to shift much of the teaching programme to online self-directed study using a large bank of resources which were shared across many universities’ (Respondent 12).

### Changes to undergraduate education due to the MLA

Six (26%) medical schools reported implementing no changes to their curriculum in response to the pending introduction of the MLA for the academic year 2024–2025. Nine (39%) medical schools reported that curriculum changes had ‘not yet’ been made and/or described currently mapping their curricula to ensure its alignment with the MLA; meanwhile, ten respondents described specific curriculum changes that they had introduced.

Examples of curriculum changes included: using more case-based learning, delivering psychiatry modules earlier in medical courses, increasing the quantity of teaching, scheduling more self-directed learning, more integration of psychiatry into general practice (GP) teaching, more teaching about specific topics such as safeguarding, implementing more workplace-based assessments (WPBAs), using more ‘single best answer’ (SBA) questions in assessments and adjusting the time allowed for answering examinations questions to mirror the timings of the MLA.

## Discussion

This first national survey of UK undergraduate psychiatry placements in over a decade reveals that there have been few significant changes, especially in placement structures and content. Crucially, very little has changed in terms of the length of psychiatry placements or the variability of placement opportunities across the nation. A few uniformities have persisted across medical schools, including core curriculum content, methods of assessment and the continued use of psychiatry in-patient wards as a core placement for medical students.

The total duration of the main psychiatry placement across the undergraduate curriculum varied significantly across the medical schools, from 4 to 12 weeks. This is similar to the 4 to 11 weeks of psychiatry placement reported in a national survey of UK medical schools in 2009.^7^ Placements should be of sufficient duration for undergraduates to observe changes in patient presentation and improvement in mental state over time, which can positively influence their views of psychiatry.^16^ Longer placements may help undergraduates to feel better embedded into clinical teams.^16^ An international survey of over 2000 medical undergraduates in 2010–2011 found no statistically significant association between the duration of psychiatry teaching and likelihood of choosing a career in the specialty; but greater exposure to psychiatry teaching correlated with improved attitudes towards psychiatry, as measured by the 18-item Attitude to Psychiatry Scale (ATP-18).^17^

Broad variation was demonstrated in the availability of optional psychiatry modules and enrichment activities across medical schools. Involvement in enrichment activities is positively associated with choosing a career in psychiatry,^17^ but such findings are prone to self-selection bias. In a 2018 survey by the RCPsych, 26% of medical undergraduates reported undertaking a special study module or elective in psychiatry, 77% of whom felt that it increased their interest in psychiatry.^8^ Involving undergraduates in extra-curricular psychiatry opportunities near the start of their degree programme can dispel any negative preconceptions they may have about the specialty. For example, extra-curricular shadowing of core trainees in psychiatry has been found to promote and sustain positive attitudes towards psychiatry among first-year medical undergraduates.^15^

The integration of psychiatry teaching with other specialties in the undergraduate curriculum was common across the 27 medical schools and has seemingly become more common since the last national survey of psychiatry curricula in 2009.^7^ In the current study, 79% of the medical schools reported integrating child and adolescent psychiatry teaching into paediatric placements, compared with 19.2% in the previous national survey.^7^ These integrated teaching methods may help undergraduates to better understand multi-morbidity and the interface between physical and mental health, enhancing their abilities to provide holistic care. They may also reinforce the need to recognise and manage acute mental health issues in all healthcare settings, as expected by the GMC.^18^ A cross-sectional survey of two medical undergraduate cohorts at one UK medical school found that undergraduates who experienced more integrated teaching of psychiatry with other specialties held improved attitudes towards psychiatry, psychiatric patients and careers in the specialty.^19^

Exposure to different psychiatric subspecialties can enhance undergraduate knowledge of psychiatry, positively influence undergraduates’ views of the specialty and demonstrate the range of opportunities afforded by a psychiatric career.^8^ There were large discrepancies in the coverage of psychiatric subspecialties across and within the different medical schools in our survey. Some respondents suggested that this was due to students undertaking placements in different hospitals, which offered different clinical services; however, online teaching and extracurricular events could be utilised to make specific experiences accessible to larger audiences. This variation of exposure was similarly reported in the earlier national survey of UK medical schools in 2009 and is consistent with qualitative reports of variable undergraduate experiences of psychiatry among psychiatry trainees.^7,16^ Other studies have reported similar differences in exposure to eating disorder and perinatal psychiatry teaching across UK medical schools.^20,21^ Quality assurance systems are vital to ensure that high standards of psychiatry teaching, support and learning opportunities are offered across different placement sites.^19^ UK medical schools should explore opportunities for undergraduates to spend time at alternative locations if specific subspecialty experiences are not available at their individual placement site.

Clinical academics and National Health Service (NHS) psychiatrists remain the most frequently involved professionals in delivering undergraduate psychiatry teaching in the UK, but there are growing opportunities for teaching to be delivered by lived experience educators (patients and/or carers) and non-medical members of the multidisciplinary team, such as pharmacists and psychologists. Involvement of lived experience educators in psychiatric education can lead to reduced stigma, greater empathy and improved understanding of holistic care among medical undergraduates.^22–24^

The reported significant shift to online modalities to deliver teaching during the COVID-19 pandemic similarly occurred in other medical specialties and has been reported previously.^25^ Key benefits of online teaching include improved flexibility and convenience of accessing learning, opportunities to involve speakers from diverse locations and the promotion of digital literacy.^25,26^ On the other hand, potential disadvantages of virtual learning include reduced socialisation among faculty and undergraduates; challenges teaching non-verbal communication; reduced opportunities to see acutely unwell patients and observe the functioning of multidisciplinary teams; and technological problems impeding learning.^25,26^ Some of the medical schools in our survey described shortening their psychiatry placements during the pandemic, but the majority did not. Similarly, a recent study reported that two-thirds of medical schools made no alterations to the duration of psychiatry modules during COVID-19, while 12.5% increased and 20.8% reduced them.^27^ Some of our surveyed medical schools expressed plans to implement blended teaching models, combining face-to-face and virtual learning post-pandemic. This is echoed in other research, where 87.5% of medical schools reported long-term changes to psychiatry teaching persisting beyond the pandemic.^27^ Online psychiatry teaching and virtual placement activities, given their scalability, may support the planned expansion of medical undergraduate numbers in the UK by increasing teaching capacity.^28^

The MLA should standardise undergraduate medical curricula since UK medical schools will be required to ‘map’ their curriculum to this examination. This could reduce variation in undergraduate experiences of psychiatry across UK medical schools. No medical schools in our survey reported removing from their curriculum psychiatry content that falls outside the scope of the MLA; however, concerns exist that learning and teaching of such topics could become de-prioritised.^29^ Collaboration and sharing of ‘best practice’ between institutions could standardise and improve undergraduate experiences of psychiatry, alongside following the RCPsych guidance for medical schools. Improving the quality of undergraduate psychiatry education is imperative in order to build a skilled workforce and positively influence patient outcomes.

### Strengths and limitations

To our knowledge, this is the largest survey of undergraduate psychiatry teaching conducted in the UK in the past decade. The survey response rate was high, with over three-quarters of eligible UK medical schools participating, increasing the generalisability of the study's findings. Newer UK medical schools were excluded from the study sample, however, and it is therefore unclear whether the findings are generalisable to these institutions. The inclusion of open and closed questions in the survey permitted both quantitative and qualitative data to be collected, but completion of open questions was variable. There were no specific questions about the design of longitudinal psychiatry placements or spiral curricula where psychiatry is taught in multiple years, although integration of psychiatry teaching with other specialties was explored. For example, the question exploring teaching topics in psychiatry focused on the main psychiatry block and did not ask specifically about integrated curricula. Moreover, the survey did not ask specifically about the existence of ‘PsychSocs’ at each organisation but enquired generally about the availability of extra-curricular modules. It is therefore possible that some aspects of psychiatry teaching, assessment and student experiences were under-reported. The cross-sectional nature of the survey precluded detailed examination of longitudinal curriculum changes over time, but comparison with a previous similar survey was possible. The surveys were completed by the undergraduate psychiatry leads at each medical school, but respondents were specifically asked whether they had formally mapped their curricula; furthermore, their responses may have been biased towards their own individual views and teaching experiences.

### Future research

Future research should explore how UK undergraduate psychiatry teaching and assessment continue to evolve as we emerge from the COVID-19 pandemic and as the MLA is formally rolled out in the future. Furthermore, the curriculum of the newer UK medical schools, once established, should be analysed and compared with existing UK medical schools to identify any discrepancies and areas of emerging innovative practice. Undergraduate perceptions of methods of delivery of psychiatry teaching and assessment should also be sought and compared with those of educators to determine whether there are differences. The impact of variation in the amount and delivery of psychiatry teaching across UK medical schools should also be evaluated to determine whether this predicts variations in undergraduate performance in the specialty and/or the MLA, as well in the number of undergraduates who subsequently enter specialist training in psychiatry.

## Supporting information

Sharma et al. supplementary material 1 Sharma et al. supplementary material

Sharma et al. supplementary material 2 Sharma et al. supplementary material

## Data Availability

The data that support the findings of this study are available from the corresponding author on request.

## About the authors

**Deepika Sharma** is a psychiatry foundation fellow at Princess Alexandra Hospital NHS Trust, Harlow, UK. **Thomas Hewson**, is an academic clinical fellow in the Postgraduate Medical Education Department, University of Manchester, UK, and core psychiatry trainee with Pennine Care NHS Foundation Trust, Ashton-under-Lyne, UK. **Sridevi Sira Mahalingappa** is a consultant psychiatrist with South London and Maudsley NHS Foundation Trust, London, UK, and an Assistant Professor in the School of Education at the University of Nottingham, UK. **James Fallon** is a clinical senior lecturer in medical education at Brighton and Sussex Medical School, Brighton, UK. **Declan Hyland** is Deputy Clinical Director for Year 4 and Specialty Lead for General Adult Psychiatry at Liverpool Medical School, UK. **Seri Abraham** is a consultant psychiatrist with Pennine Care NHS Foundation Trust, Ashton-under-Lyne, UK. **Alexa Sidwell** is a clinical nurse educator with Derbyshire Healthcare Foundation Trust, Derby, UK. **Subodh Dave** is a consultant psychiatrist with Derbyshire Healthcare Foundation Trust, Derby, UK, Professor of Psychiatry at the University of Bolton, UK, and Dean of the Royal College of Psychiatrists, London, UK.
