# Supplementary material for: Teaching, assessment and best practice in undergraduate psychiatry education in the UK: cross-sectional survey
Source: BJPsych Bull. 2024 Dec;48(6):377–83. doi: 10.1192/bjb.2024.2 (PMC11669459; doi:10.1192/bjb.2024.2)
Supplement: Sharma et al. supplementary material 1 — Sharma et al. supplementary material [file S2056469424000020sup001.docx]

**Appendix 1: Full list of survey questions

Undergraduate Psychiatry Survey**

**Start of Block: Block 1**

If you continue to the survey, you confirm that:

 1.  You have read and understood the information on the previous page.

 2.  You understand that your participation is voluntary, and I can end the survey at any time and withdraw my data by closing the browser.

 3.  You understand that your answers are confidential and you/your institution will not be identifiable in any report or publication.

 4.  You understand the overall anonymized data from this study may be used in the future for research (with research ethics approval) and teaching purposes.

Q1 I confirm that I have read, understood and agree with the 4 statements above.

- Yes (1)
- No (2)

**End of Block: Block 1**

**Start of Block: Block 2**

**Section 1:  About You**

Q2 **1.  Please choose the medical school you are attached to.**

▼ Drop down menu

Q3 **2.  What medical degrees does your university offer? Tick all that apply.**

- 4 year graduate-entry level course (1)
- 5 year undergraduate course (2)
- 5 year undergraduate course with intercalated degree/BMedSci (3)
- 6 year undergraduate course with intercalated degree (4)
- 6 year undergraduate course with foundation year (5)
- Other (6)

**End of Block: Block 2**

**Start of Block: Block 3**

**Section 2:  Psychiatry placements**

Q4 **1.  How many weeks of clinical placement are offered in the main psychiatry module (not including any optional modules or aspects of psychiatry covered in other modules)? Slide bar to correct number of weeks.**

Q5 **2.  What range of teaching methods are used to deliver teaching during the psychiatry placement?  Please state the estimated total number of hours delivered of each method over the whole placement.**

Q6 **3.  Who has an appointed teaching role to deliver psychiatry teaching at your university/trust? (Tick all that apply).**

- Clinical academics (1)
- NHS Psychiatrists (2)
- General Practitioners (3)
- Other primary care staff (4)
- Advanced clinical practitioners (5)
- Nursing staff (6)
- Psychologists (7)
- Pharmacists (8)
- Occupational therapists (9)
- Physician Associates (10)
- Patient as educators/ lived-experience workers (11)
- Carers (12)
- Non-clinical staff (13)
- Other NHS staff (14)
- Non-NHS staff (15)
- Voluntary sector staff (16)
- Other (17)

Q7 **4.  What content is formally covered during your mandatory psychiatry placement?  Tick all that apply.**

- Addiction psychiatry (1)
- Anxiety disorders (2)
- Ethnicity and mental health (3)
- Child and adolescent psychiatry (4)
- Communication skills – de-escalation (5)
- Communication skills – handover (6)
- Communication skills – psychologically distressed patients (7)
- Confidentiality (8)
- Dementia (9)
- Depression (10)
- Derealisation and depersonalisation (11)
- Dual diagnosis – substance abuse (12)
- Eating disorders (13)
- Forensic psychiatry (14)
- History taking (15)
- Intellectual disability (16)
- Liaison psychiatry (17)
- Managing common psychiatric emergencies (mental health) (18)
- Managing common psychiatric emergencies (physical health) (19)
- Mental Capacity Act/ Adults with Incapacity Act (Scotland)/ Assisted Decision Making Capacity Act (Ireland) (20)
- Perinatal mental health (21)
- Psychopharmacology (22)
- Mental Health Act (23)
- Mental state examination (24)
- Mood disorders (25)
- Neuropsychiatry (26)
- Older adult mental health (27)
- Personality disorders (28)
- Post-traumatic stress disorder (29)
- Promoting psychiatry as a career (30)
- Professionalism (31)
- Psychosexual disorders (32)
- Psychotic disorders (33)
- Public health psychiatry (34)
- Risk assessment (35)
- Self-harm (36)
- Sleep disorders (37)
- Social justice/deprivation (38)
- Spirituality/culture in psychiatry (39)
- Stigma towards mental illness (40)
- Technology/digital advances in psychiatry (41)
- Using the Mental Health Act in practice (42)

Q8 **5.  As best you can please describe the structure/timetable of a typical clinical psychiatry placement offered to undergraduate medical students?**  
 *Prompts:-  What experiences are offered to students in their psychiatry placement (e.g. CMHT, inpatient, older adults, other specialties, 3rd sector). How are students taught required knowledge, skills and attitudes and how are these assessed?* *How does placement provision differ between hospital sites?*

________________________________________________________________

Q9 **6.  Do you offer training specifically in psychotherapy competencies/reflective practice as part of the psychiatric placement? (e.g. Balint Groups, Schwartz Rounds).  Please tick the most appropriate option(s).**

- Balint groups (1)
- Schwartz rounds (2)
- Reflective practice sessions (3)
- Other (4)

**End of Block: Block 3**

**Start of Block: Block 4**

**Section 3:  Psychiatry teaching during medical school**

Q10 **1.  How many hours are spent on mandatory psychiatry teaching each year of medical school (not including optional modules)?**

|  | 0 hours (1) | <2 hours (2) | 3-5 hours (3) | 6-10 hours (4) | 11-20 hours (5) | >20 hours (6) |
| --- | --- | --- | --- | --- | --- | --- |
| Year 1 (1) |  |  |  |  |  |  |
| Year 2 (2) |  |  |  |  |  |  |
| Year 3 (3) |  |  |  |  |  |  |
| Year 4 (4) |  |  |  |  |  |  |
| Year 5 (5) |  |  |  |  |  |  |
| Year 6 (6) |  |  |  |  |  |  |

Q11 **What is covered in during mandatory teaching of psychiatry during each year of medical school?  Please give as much detail as possible.**

________________________________________________________________

Q12 **2.  Is psychiatry integrated with any other specialties during the course?**Please estimate the number of hours of integrated teaching given.  
 *Examples of integrated teaching:- CAMHS teaching during paediatric module/placement, postnatal depression during women's health, old age psychiatry during elderly care module/placement.*  

|  | 0 hour (1) | <1 hour (2) | 1-2 hours (3) | 3-4 hours (4) | 4-5 hours (5) | 5-6 hours (6) | >6 hours (7) |
| --- | --- | --- | --- | --- | --- | --- | --- |
| Women's Health (1) |  |  |  |  |  |  |  |
| Child Health (2) |  |  |  |  |  |  |  |
| General Practice (3) |  |  |  |  |  |  |  |
| Elderly Care (4) |  |  |  |  |  |  |  |
| Neurology (5) |  |  |  |  |  |  |  |
| Cardiology (6) |  |  |  |  |  |  |  |
| Respiratory (7) |  |  |  |  |  |  |  |
| Hepatobiliary (8) |  |  |  |  |  |  |  |
| Gastroenterology (9) |  |  |  |  |  |  |  |
| Surgery/Anaesthetics (10) |  |  |  |  |  |  |  |
| A&E (11) |  |  |  |  |  |  |  |
| GU Medicine (12) |  |  |  |  |  |  |  |
| Other (13) |  |  |  |  |  |  |  |

Q13 **3.  Does your university offer optional psychiatry-related modules during the undergraduate course?  Please describe the optional modules offered.**

________________________________________________________________

**End of Block: Block 4**

**Start of Block: Block 5**

**Section 4:  Assessment**

Q14 **1.  Please describe the formative and/or summative assessment processes for students, in order to successfully complete their psychiatry placements?**

________________________________________________________________

Q15 **2.  Please choose the summative assessment method(s) used at your university for psychiatry throughout medical school.  Tick all appropriate options.**

- Short answer (SAQs) (1)
- Multiple choice questions (MCQs) (2)
- Extended matching items/questions (EMI/EMQ) (3)
- Placement-based assessment (4)
- Objective structured clinical examination (OSCE) (5)
- Case-based problem solving (6)
- Essay (7)
- Project/poster/presentation (8)
- Portfolio (9)
- Reflection (10)
- Other (11)

**End of Block: Block 5**

**Start of Block: Block 6**

**Section 5:  COVID-19 teaching and future plans**

Q16 **1.  What changes have you made to your curriculum during the COVID-19 pandemic to deliver psychiatric teaching?**

________________________________________________________________

Q17 **2.  What online learning platforms do you currently use to deliver psychiatry teaching (post-COVID)?**

________________________________________________________________

Q18 **3.  What online assessment platforms do you use for psychiatry (post-COVID)?**

________________________________________________________________

Q19 **4.  Have you made any changes to your curriculum in preparation for the upcoming medical licensing exam (MLE)?  If so please describe changes made.**

________________________________________________________________

**End of Block: Block 6**

**Start of Block: Block 7**

**Section 6:  Follow-up Interviews**

Q20 **We are conducting follow-up interviews via MS Teams to help us clarify answers to this survey.  Are you happy for one of our researchers to get in touch with you to organise a time for this?**

- Yes, I'm happy to be interviewed (1)
- No, I'd rather not be interviewed (2)

**End of Block: Block 7**
