# Supplementary material for: Teaching, assessment and best practice in undergraduate psychiatry education in the UK: cross-sectional survey
Source: BJPsych Bull. 2024 Dec;48(6):377–83. doi: 10.1192/bjb.2024.2 (PMC11669459; doi:10.1192/bjb.2024.2)
Supplement: Sharma et al. supplementary material 2 — Sharma et al. supplementary material [file S2056469424000020sup002.docx]

**Appendix 2**

*Table 2: Educational methods used to teach psychiatry across 27 UK medical schools*

| Teaching Method | Number of Medical Schools Spending X Amount of Time on Each Teaching Method During Psychiatry Placements | | | | | | Median amount of time spent on each teaching method |
| --- | --- | --- | --- | --- | --- | --- | --- |
|  | 0 hours | 1-2 hours | 3-5 hours | 6-10 hours | 11-20 hours | >20 hours |  |
| Small group teaching | 0 | 0 | 6 | 6 | 8 | 7 | **11- 20 hours** |
| Lectures | 3 | 1 | 3 | 7 | 8 | 5 | **6-10 hours** |
| E-learning | 4 | 3 | 3 | 7 | 5 | 4 | **6-10 hours** |
| Case-based learning | 4 | 1 | 10 | 8 | 2 | 2 | **3-5 hours** |
| Simulation or role play | 6 | 3 | 11 | 3 | 2 | 1 | **3-5 hours** |
| Workshops | 8 | 0 | 4 | 5 | 3 | 0 | **3-5 hours** |
| Expert patient sessions | 8 | 8 | 7 | 2 | 1 | 0 | **1-2 hours** |
| Student-led teaching | 14 | 2 | 4 | 3 | 1 | 0 | **0 hours** |
| Journal clubs | 17 | 4 | 3 | 1 | 0 | 0 | **0 hours** |
| Film clubs | 19 | 4 | 2 | 0 | 0 | 0 | **0 hours** |
| Webinars (pre-COVID curriculum only) | 20 | 1 | 2 | 0 | 0 | 0 | **0 hours** |
| Virtual reality | 21 | 1 | 1 | 0 | 1 | 0 | **0 hours** |
| Book clubs | 22 | 3 | 0 | 0 | 0 | 0 | **0 hours** |
| Gaming | 22 | 0 | 1 | 0 | 0 | 0 | **0 hours** |
